# The mitochondrial dysfunction, alongside the modifiable burden of traditional risk factors, drives the development of early-onset coronary artery disease

**DOI:** 10.3389/fcvm.2025.1538202

**Published:** 2025-02-19

**Authors:** Jonica Campolo, Paola Canale, Gianluca Gazzaniga, Marina Parolini, Emanuela Piccaluga, Irene Bossi, Jacopo Oreglia, Andrea Borghini, Irene Marinaro, Maria Grazia Andreassi

**Affiliations:** ^1^ CNR Institute of Clinical Physiology, ASST Grande Ospedale Metropolitano Niguarda, Milan, Italy; ^2^ CNR Institute of Clinical Physiology, Pisa, Italy; ^3^ Health Science Interdisciplinary Center, Sant’Anna School of Advanced Studies, Pisa, Italy; ^4^ Department of Medical Biotechnology and Translational Medicine, Postgraduate School of Clinical Pharmacology and Toxicology, Università degli Studi di Milano, Milan, Italy; ^5^ Department of General Surgery and Surgical Specialty Paride Stefanini, Sapienza University of Rome, Rome, Italy; ^6^ Interventional Cardiology, ASST Grande Ospedale Metropolitano Niguarda, Milan, Italy

**Keywords:** mitochondrial dysfunction, atherosclerosis, premature vascular ageing, early coronary artery disease, mtDNA-CN, mtDNA^4977^

## Abstract

**Objective:**

Mitochondrial dysfunction is associated with increased risk of atherosclerosis by disrupting key cellular processes that contribute to premature vascular ageing. However, the specific role of mitochondrial dysfunction in early-onset coronary artery disease (EOCAD), which is increasing at a particularly alarming rate, remains largely unexplored. This study investigated the association of leukocyte mtDNA-CN and mtDNA^4977^ deletion with the risk of EOCAD.

**Methods:**

The study included 118 patients (99 men, 51.0 ± 5.6 years) with angiographically EOCAD (≤60 years) and 150 healthy controls (94 men, 49.8 ± 5.8 years). Quantitative RT-PCR was used to quantify mtDNA-CN and mtDNA^4977^ deletion rate.

**Results:**

The EOCAD group had a higher prevalence of male gender (*p* < 0.001), smoking (*p* = 0.001), hypertension (*p* < 0.001), diabetes mellitus (*p* = 0.04) and obesity (*p* < 0.001) than controls. EOCAD patients had lower mtDNA-CN (*p* < 0.001) and higher mtDNA^4977^ deletion (*p* = 0.026). Low mtDNA-CN levels were significantly associated with male gender (*p* < 0.001), smoking (*p* = 0.004), hypertension (*p* = 0.039), hypercholesterolemia (*p* < 0.001), and obesity (*p* < 0.001). Increased levels of the mtDNA^4977^ deletion were significantly higher in males (*p* = 0.026) and hypercholesterolemic patients (*p* < 0.001). The ROC curve of mtDNA-CN and mtDNA^4977^ deletion in predicting EOCAD showed an AUC of 0.902 (95% CI 0.867–0.937, *p* < 0.001) and 0.762 (95% CI 0.691–0.834, *p* < 0.001), respectively. Logistic regression analysis adjusted for confounders showed that both mtDNA-CN and mtDNA^4977^ deletion were independent significant predictors of EOCAD (*p* < 0.001 and *p* = 0.001, respectively).

**Conclusions:**

EOCAD is characterized by a high prevalence of modifiable risk factors and mitochondrial damage, underscoring the need for more efforts to reduce the burden of traditional risk factors and highlighting the potential for innovative mitochondrial-targeted therapies.

## 1 Introduction

Coronary artery disease (CAD) remains a leading cause of illness, disability, and mortality worldwide, despite advances in prevention and treatment strategies (1). Alarmingly, recent trends have shown a rise in premature coronary artery disease, also called early-onset CAD (EOCAD), among younger populations (2, 3). Individuals with EOCAD often exhibit a high prevalence of modifiable traditional risk factors such as dyslipidemia, smoking, diabetes mellitus, hypertension, and overweight/obesity (4–8). Non-classical risk factors including inflammation, infections, sleep deprivation, and exposure to air pollution and environmental noise also warrant consideration (1, 9).

Moreover, it is well known that premature CAD is characterized by a rapid progression of the disease and is associated with a poorer long-term prognosis (10, 11). Thus, further research is essential to better understand the risk profile, behaviors, and underlying biological mechanisms contributing to premature CAD in younger individuals (12, 13).

Interestingly, recent research has identified a significant association for the mitochondrial mtDNA 16223T variant and heteroplasmy with an increased presence of cardiovascular risk factors and premature CAD (14).

Indeed, several studies have highlighted the direct role of mtDNA-mediated mitochondrial dysfunction in the initiation and progression of atherosclerosis (15), and the importance of preserving the mitochondrial genome to maintain healthy vessel and to delay vascular aging (16).

Mitochondria, essential cellular organelles, are primarily responsible for energy production through ATP generation via oxidative phosphorylation. They also play key roles in various cellular processes, including modulation of oxidation–reduction status, regulation of senescence and apoptosis. Mitochondria possess their own circular genome, known as mitochondrial DNA (mtDNA), with each mitochondrion containing multiple copies of this genome, referred to as mtDNA copy number (mtDNA-CN). The mtDNA-CN is positively correlated with mitochondrial enzyme activity and ATP production, making it a surrogate biomarker for assessing mitochondrial function (17).

Lower mtDNA-CN has shown associations to several aging-related diseases and all-cause mortality, including cardiovascular disease (18).

Additionally, mtDNA is particularly vulnerable to damage from reactive oxygen species (ROS) due to the absence of histones and a less efficient repair mechanism. Besides rare pathogenic variants, the accumulation of mutations in the mitochondrial genome can further accelerate the development of atherosclerosis (19). Among various mtDNA defects, a large-scale deletion of 4,977 bp, known as the mtDNA^4977^ common deletion, results in the loss of nearly one-third of the mitochondrial genome. This leads to the accumulation of dysfunctional mitochondrial transcripts and a consequent increase in ROS production (20).

In light of this, we hypothesized that reduced mtDNA integrity and mitochondrial dysfunction may predispose individuals to accelerated atherosclerosis and the premature development of CAD. To test this hypothesis, we investigated the association of leukocyte mtDNA^4977^ deletion and mtDNA-CN with early-onset coronary artery disease.

## 2 Materials and methods

### 2.1 Study population

The study employed a case-control design, involving 118 patients (99 males, mean age 51.0 ± 5.6 years) with EOCAD (≤60 years) recruited from the GENOCOR (Genetic Mapping for Assessment of Cardiovascular Risk) and VICTORIA (Vascular Senescence and Atherosclerotic Plaque Vulnerability) databases (21, 22). Cases included patients with angiographically confirmed CAD, defined by significant coronary stenosis in at least one vessel (>50% lumen reduction). The severity of CAD was classified based on the number of vessels involved (one-, two-, or three-vessel disease). All subjects had a diagnosis of stable angina pectoris or acute coronary syndrome (myocardial infarction or unstable angina). Exclusion criteria comprised patients with known malignancy, end-stage renal disease, or other severe illnesses that could confound study outcomes.

A control group comprised 150 healthy individuals (94 males, mean age 49.8 ± 5.8 years), who were members of the medical and technical staff at our institution and exhibited no clinical evidence of cardiovascular disease. Classic cardiovascular risk factors were recorded for all participants, including age, gender, diabetes (fasting plasma glucose >120 mg/dl or on glucose-lowering medication), hypercholesterolemia (plasma cholesterol >220 mg/dl or on lipid-lowering therapy), obesity (body mass index >30 kg/m^2^), and arterial hypertension (systolic blood pressure >140 mmHg and/or diastolic pressure >90 mmHg, or on antihypertensive therapy). Smoking status was categorized as current smokers (at least 3 cigarettes per day), past smokers (abstinent for at least 6 months), and non-smokers (never smoked). For analysis, smoking patients included both past and current smokers.

### 2.2 Analysis of mtDNA^4977^ deletion and mtDNA-CN

Blood samples were taken from participants on admission, prior to any diagnostic or therapeutic procedure, and DNA was extracted using established protocols (23, 24).

The mtDNA-CN and mtDNA^4977^ deletion levels were quantified using quantitative real-time PCR (CFX384 Touch Real-time PCR detection system, Bio-Rad, Hercules, CA, USA).

To determine the levels of the mtDNA^4977^ deletion (spanning nucleotides 8,470–13,447 bp), we amplified the NDI1 gene located in an undeleted region of mtDNA (mtNDI1) along with the remaining fragment after the mtDNA^4977^ deletion. The difference in the average threshold cycle (Ct) values was used to measure the relative content. The percentage of the mtDNA^4977^ deletion was calculated as follows: 2^–ΔCt^ × 100%, where ΔCT = Ct × mtDNA^4977^−Ct × mtNDI1. Additionally, ΔCt values were computed as the difference between the Ct of the ß-globin gene and the Ct of the NDI1 gene, which was used to measure mtDNA-CN relative to genomic DNA (gDNA). mtDNA-CN was then calculated by using (2^-ΔCt^) method where ΔCt = Ct mtNDI1—Ct gDNA. Each sample was run in triplicate to ensure intra-assay precision. Because there were insufficient amounts of DNA for some participants, we had measurements mtDNA-CN on 149 controls and 117 cases, and measurements on mtDNA deletion on 119 controls and 93 cases.

### 2.3 Statistical analyses

Values are presented as mean ± standard deviation (SD), median with interquartile range (IQR) or percent, according to the nature of the data.

Categorical data, expressed as frequencies and percentages, were compared using the Chi-square or Fisher's exact test when appropriate, while continuous variables using the Student's *t*-test and Mann–Whitney *U* test for parametric and non-parametric data, respectively.

The association between mtDNA^4977^ deletion, mtDNA-CN and other continuous parameters was assessed using Spearman rank correlation.

The receiver operating characteristic (ROC) curve was used as a measure of diagnostic accuracy of mtDNA^4977^ deletion and mtDNA-CN and the area under the curve (AUC) along with 95% confidence intervals (95% CI), was calculated to evaluate the specificity, sensitivity and accuracy for predicting EOCAD, and optimal cutoff values were determined by the Youden index.

Univariate and multivariate logistic regression analyses confined to subjects with complete dataset were also performed to determine odds ratios (ORs) and 95% CIs. mtDNA^4977^ deletion and mtDNA-CN were analyzed as continuous variables, and as categorical variables dichotomized according to the optimal cut-off values identified by ROC curves.

Age, gender, smoking, hypertension, hypercholesterolemia, diabetes, obesity were considered as potential confounders, and were therefore tested in univariate and multivariate models.

Power analysis showed that our sample size of 118 EOCAD and 150 healthy controls allowed to detect a medium effect size (*d* = 0.5) at 0.05 significance level and 80% power using G*Power software. A *p*-value of <0.05 was considered statistically significant in this study. Statistical analyses were performed using SPSS ver. 24.0 software package (IBM SPSS, New York, USA).

## 3 Results

### 3.1 Baseline characteristics of study participants

**Figure 1:**
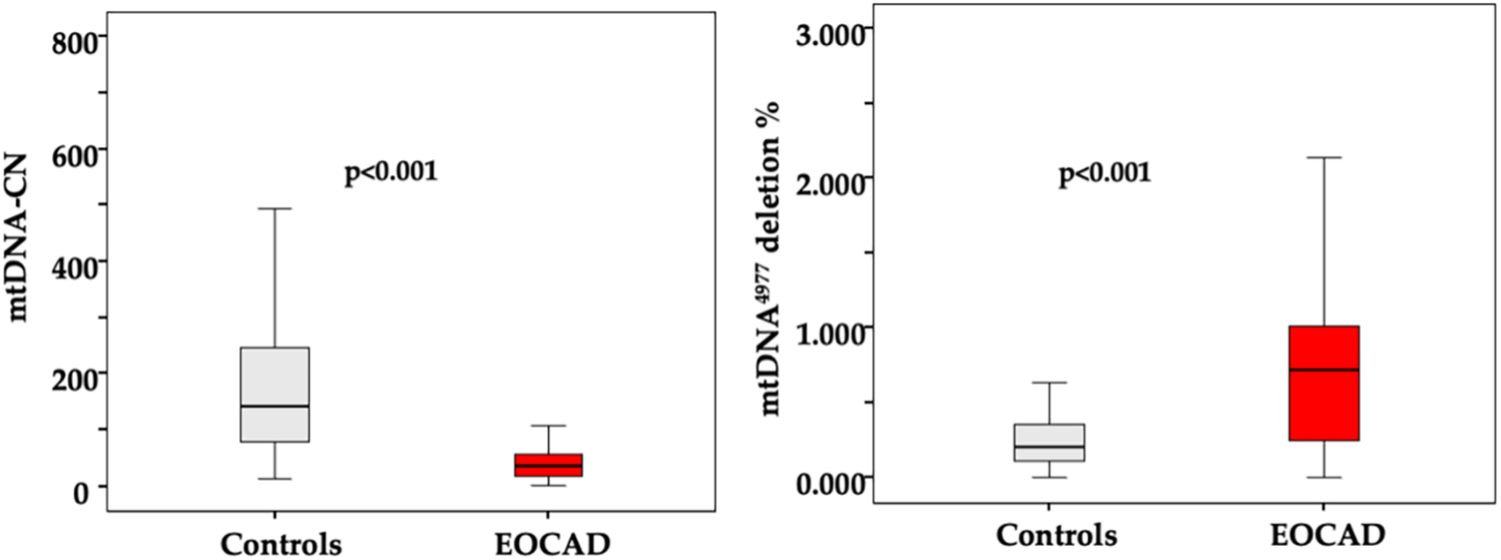
Box and whisker plot showing the levels of mtDNA CN and mtDNA^4977^ deletion in the leukocytes of the control group and the EOCAD group. Interquartile range, median, maximum and minimum values are shown in the box-and-whisker plots.

The majority of patients presented with stable angina (82%), and most had single-vessel obstructive CAD (61%). A detailed description of the study population is presented in Table 1. The average age of patients with EOCAD and controls was not significantly different. As expected, the prevalence of male gender (*p* < 0.001), smoking (*p* = 0.001), hypertension (*p* < 0.001), diabetes mellitus (*p* = 0.04) and obesity (*p* < 0.001) was significantly higher in the EOCAD group compared to healthy controls. As shown in Figure 1, mtDNA-CN levels were significantly lower in patients with EOCAD compared to controls (*p* < 0.001). Similarly, the mtDNA^4977^ deletion was higher in the EOCAD group than in the control group (*p* < 0.001).

**Table 1:** Demographic and clinical characteristics of the study population.

Characteristics	EOCAD patients	Controls	*p* value
Age (years)	51.0 ± 5.6	49.9 ± 5.8	0.102
Male, *n* (%)	99 (83.9)	94 (62.7)	0.0001
Smoking, *n* (%)	80 (67.8)	69 (46.0)	0.001
Hypertension, *n* (%)	53 (44.9)	29 (19.3)	<0.001
Hypercholesterolemia, *n* (%)	93 (78.8)	32 (21.3)	<0.001
Diabetes, *n* (%)	12 (10.2)	5 (3.3)	0.04
Obesity, *n* (%)	40 (33.9)	15 (10.0)	<0.001

Across the entire sample, there was a significant inverse correlation between mtDNA^4977^ deletion and mtDNA-CN levels (Spearman's rho = −0.372, *p* < 0.001).

### 3.2 Influence of traditional risk factors on mtDNA^4977^ deletion and mtDNA-CN

In the overall study population, mtDNA-CN and the mtDNA^4977^ deletion did not show a significant correlation with chronological age. However, in patients with EOCAD, a significant positive correlation between mtDNA-CN and age was observed (Spearman's rho = 0.321, *p* < 0.001).

**Figure 2:**
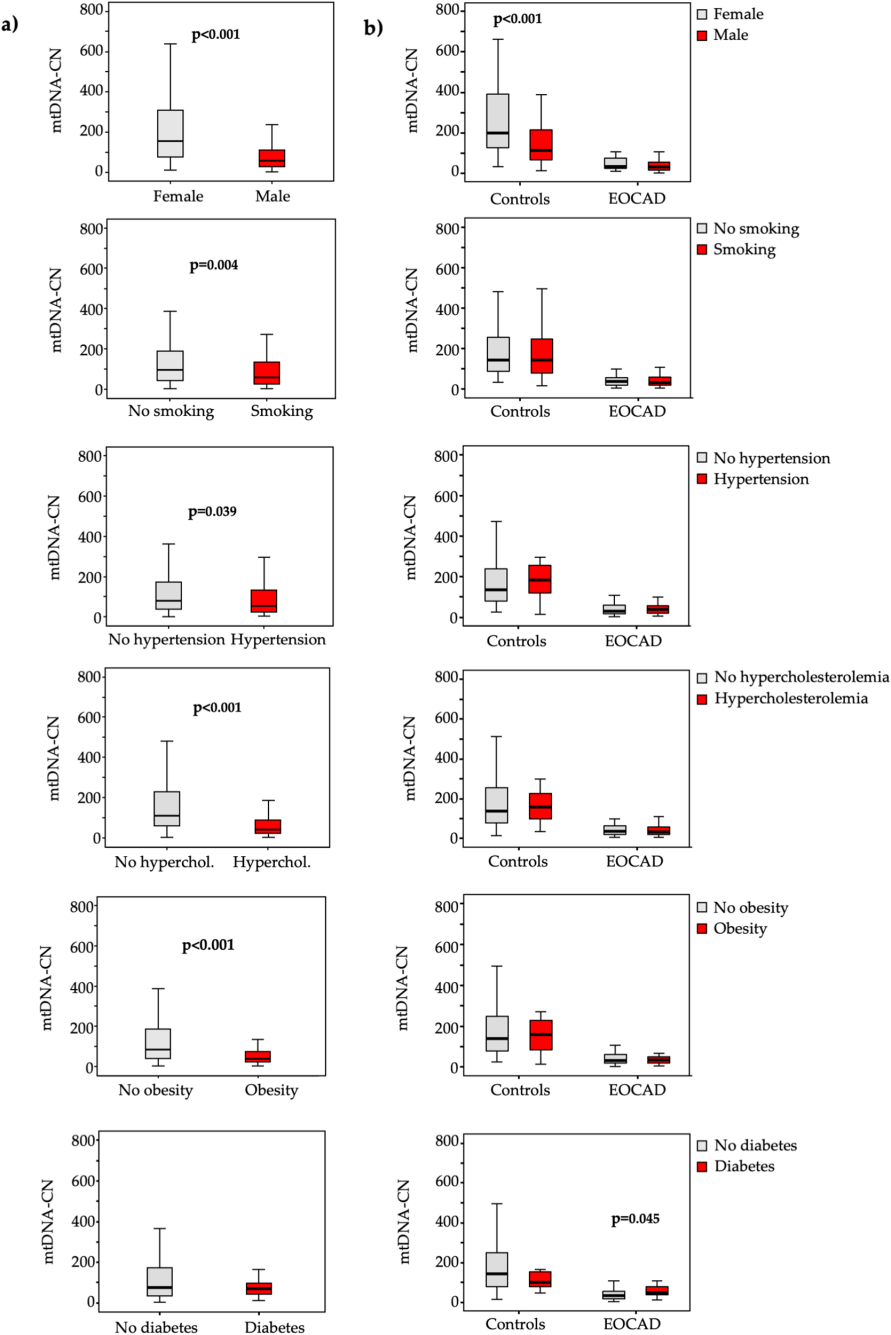
Box and whiskers plot of the mtDNA-CN in the total study population **(a)** and in each group **(b)** according to the absence (grey bars) or presence (red bars) of various traditional vascular risk factors.

**Figure 3:**
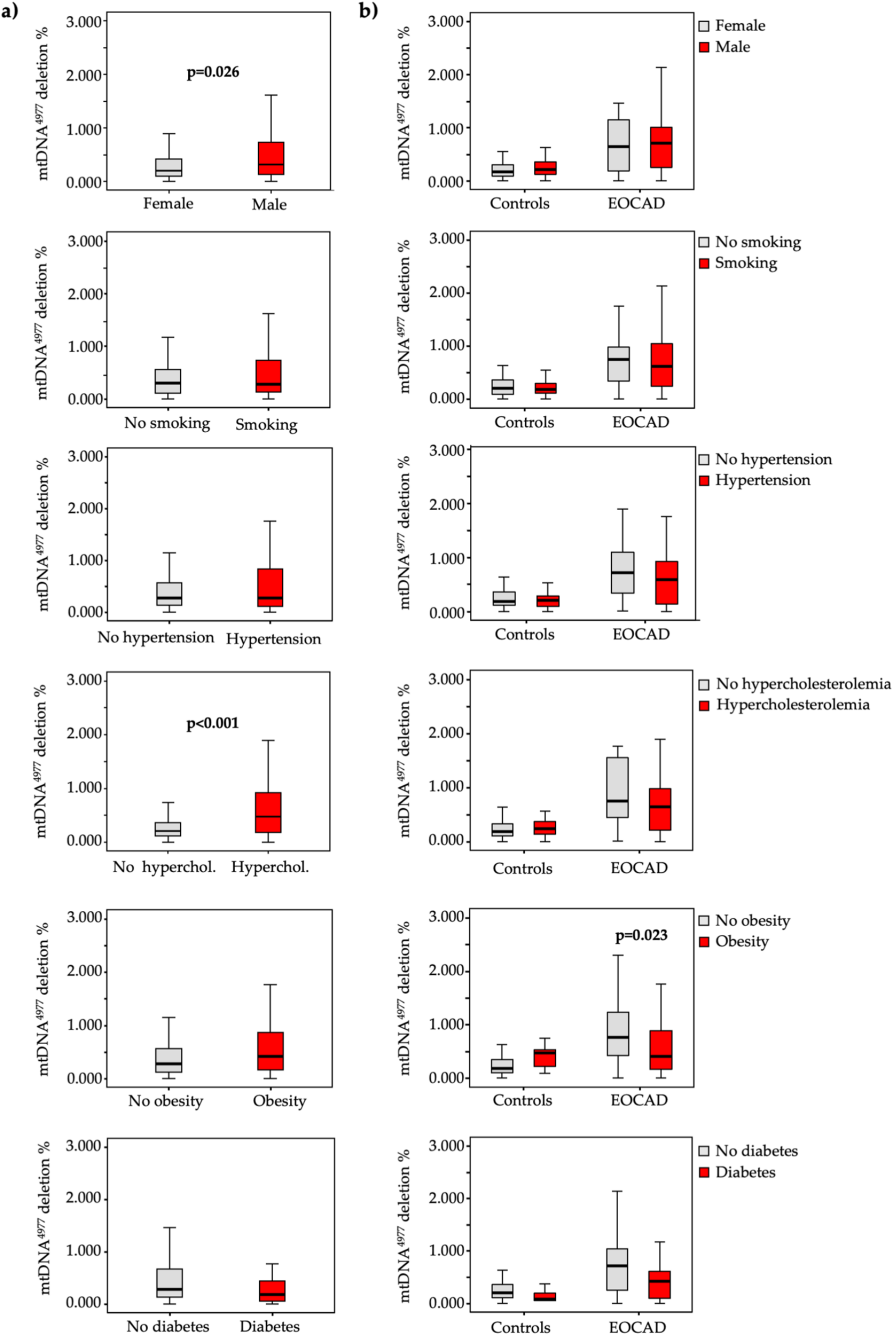
Box and whiskers plot of the mtDNA^4977^ deletion in the total study population **(a)** and in each group **(b)** according to the absence (grey bars) or presence (red bars) of various traditional vascular risk factors.

In terms of cardiovascular risk factors, lower mtDNA-CN levels (Figure 2) were significantly associated with male gender (*p* < 0.001), smoking (*p* = 0.004), hypertension (*p* = 0.039), hypercholesterolemia (*p* < 0.001), and obesity (*p* < 0.001), while higher mtDNA^4977^ deletion levels (Figure 3) were significantly higher in male gender (*p* = 0.026) and hypercholesterolemic patients (*p* < 0.001).

A similar pattern emerged when these analyses were conducted separately for cases and controls (Figures 2, 3).

No significant differences in mtDNA-CN or mtDNA^4977^ deletion were found among patients with two- or three-vessel disease compared to those with single-vessel disease.

### 3.3 Receiver operating curve (ROC) analysis

**Figure 4:**
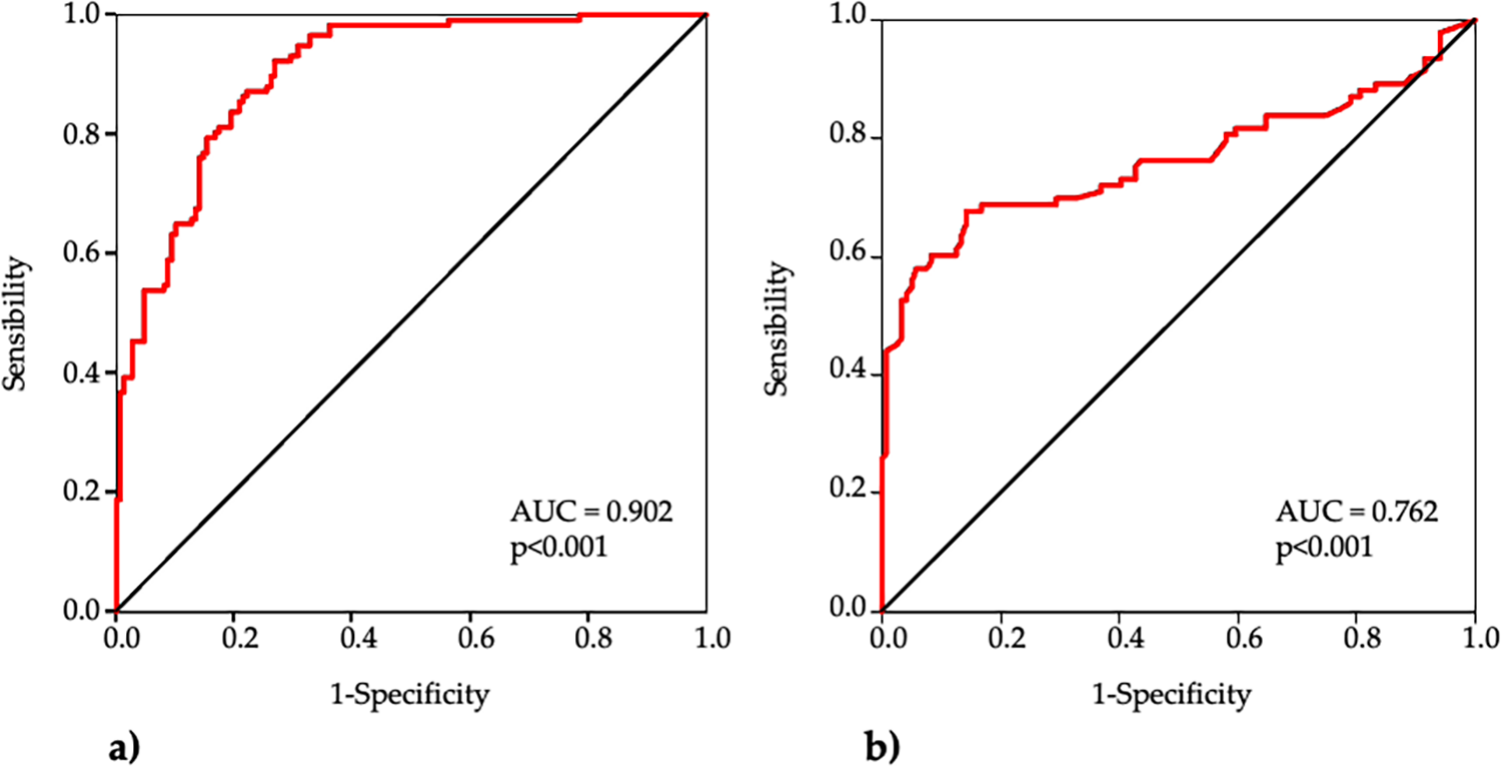
Receiver operating characteristic (ROC) curve analysis of mtDNA CN **(a)** and mtDNA^4977^ deletion **(b)** for the diagnosis of EOCAD.

ROC curve analyses indicated that each marker significantly predicted EOCAD (Figure 4).

The AUC of mtDNA-CN was 0.902 (95% CI 0.867–0.937, *p* < 0.001) and the best cut-off was <85 with sensitivity 92%, specificity 73% and accuracy 82%. The AUC of mtDNA^4977^ deletion was 0.762 (95% CI 0.691–0.834, *p* < 0.001), and the best cut-off was >0.40 with sensitivity 68%, specificity 86% and accuracy 78%.

### 3.4 Risk analysis

**Figure 5:**
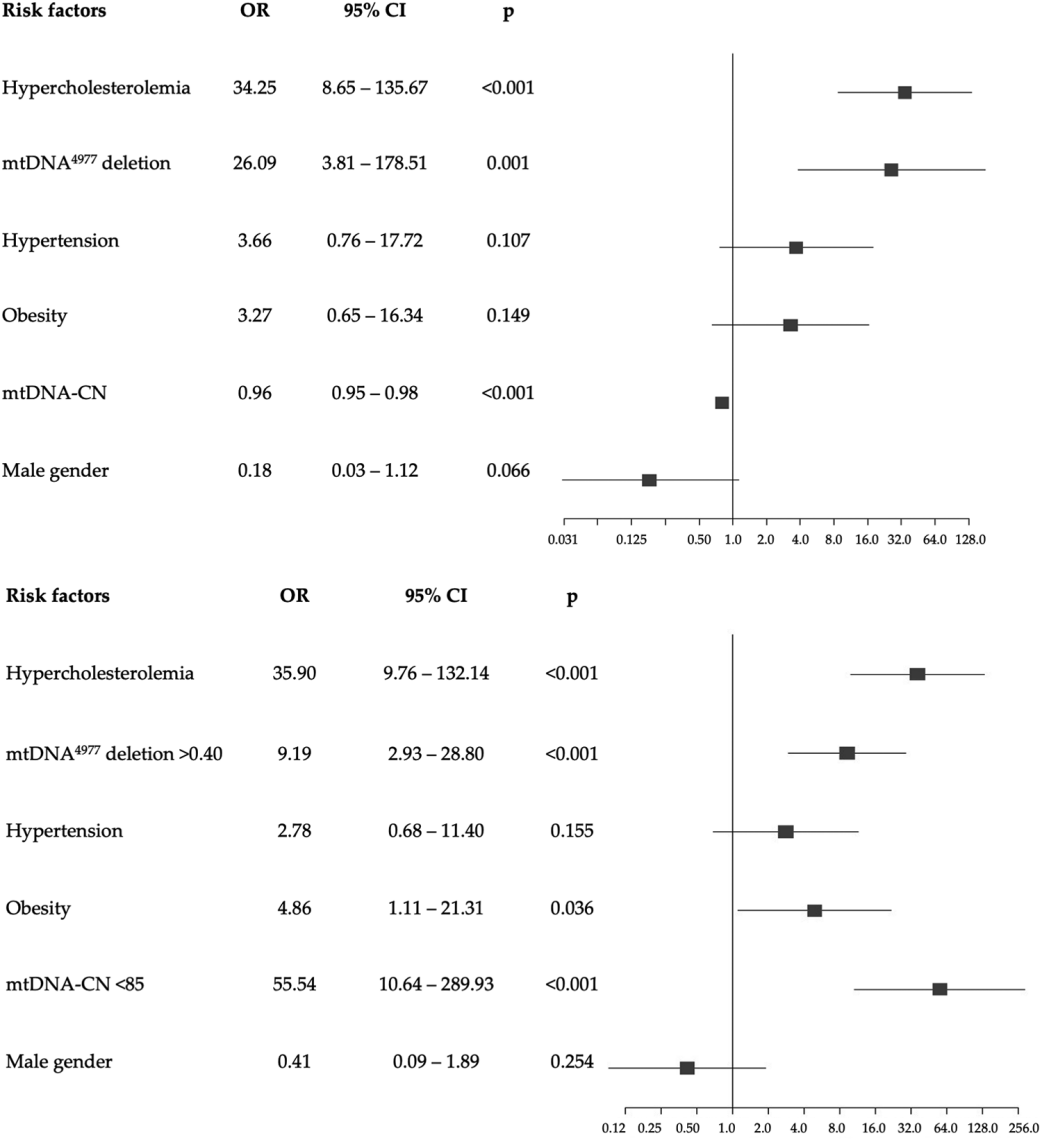
Forest plots of multivariable logistic regression analysis with odds ratio (95% confidence interval) of the association of each potential risk factor compared to none (reference) with the development of EOCAD. The top figure shows the model with the mtDNA^4977^ deletion and the mtDNA-CN as continuous variables, while the bottom figure refers to the model with categorical variables, dichotomized according to the optimal thresholds identified by the ROC curves.

Logistic regression analysis adjusted for confounders revealed that increased mtDNA-CN levels were associated to a decreased risk of developing EOCAD with an OR of 0.96 (95% CI: 0.95–0.98; *p* < 0.001). Similarly, we found an association between high levels of mtDNA^4977^ deletion and increased risk of EOCAD (OR = 26.1, 95% CI: 3.81–178.51; *p* = 0.001). We also performed a multiple logistic regression analysis using each mitochondrial biomarker dichotomized on the optimal cutoff value determined by the ROC analysis, and the results support the previous observations obtained as continuous variables (Figure 5).

## 4 Discussion

To the best of our knowledge, this is the first attempt to explore the interaction between mitochondrial damage and dysfunction in blood, and early-onset of coronary artery disease. We identified an independent, significant association between both mtDNA^4977^ deletion and mtDNA-CN and incident EOCAD after accounting for traditional risk factors. In addition, our results showed significant associations between traditional cardiovascular risk factors and mitochondrial dysfunction, suggesting that the mtDNA damage and dysfunction induced by atherosclerosis-related risk factors may play a crucial role in the early initiation and progression of atherosclerosis (25). In addition, our results agree with previous findings suggesting that mitochondrial dysfunction may have a gender-specific component influenced by hormonal and metabolic factors. In fact, women's mitochondria are better able to deal with stressful situations and conditions, and are more resistant to DNA damage and mutations (17, 18).

Previous studies have found that lower mtDNA-CN in blood is consistently associated with higher risk of CAD, reflecting mitochondrial dysfunction and oxidative stress, both of which are critical in CAD pathogenesis (26).

In particular, three large prospective studies suggested that mtDNA-CN in circulating leukocytes may have clinical utility in improving CVD risk classification (27).

Interestingly, a case-control study also found a significant joint effect between low levels of mtDNA-CN and risk factors like plasma LDL-C or smoking with increasing the incidence of CAD (28).

Moreover, cardiometabolic risk factors such as high blood pressure, obesity, and dyslipidemia have been shown to impact mtDNA-CN (29–31).

Regarding mtDNA damage, previous studies showed that mtDNA^4977^ deletion accumulates with age in normal hearts (19, 20) and that the levels of mtDNA^4977^ deletion are increased in human atherosclerotic lesions (32).

Our previous study also reported significant associations between lower mtDNA^4977^ deletion levels and poorer CVD outcomes as well as increased all-cause mortality in patients with stable CAD, confirming the critical role of mitochondrial dysfunction in atherosclerosis (24).

Remarkably, experimental studies in animal models further support the role of mtDNA damage in atherosclerosis. For instance, mtDNA damage has been observed during the early stages of atherogenesis in ApoE-/- mice and correlates with the severity of the disease (33). In double knock-out ATM+/-/ApoE-/- mice, an increased frequency of the mouse mitochondrial “common” deletion was associated with impaired respiratory complex activity (34). These mitochondrial alterations accelerated the development of atherosclerosis and were linked to key metabolic syndrome features such as hypertension, hypercholesterolemia, and obesity (35).

In response to reactive oxygen species, human vascular endothelial and smooth muscle cells accumulate more mtDNA damage than nuclear DNA, impairing key cellular processes like growth, signaling, and apoptosis. This cell dysfunction can, in turn, contribute to the initiation and progression of atherosclerotic lesions (35).

In addition, point mtDNA mutations in blood cells, especially rare variants and heteroplasmy levels (a mixture of both wild-type and mutant species of mtDNA), are being actively investigated in relation to atherosclerosis, coronary heart disease and various atherosclerosis-related diseases such as arterial hypertension and diabetes mellitus, and the list of identified mutations continues to grow (14, 36, 37). These mutations cause structural and functional changes in the mtDNA OXPHOS-related and mt-tRNA genes, which in turn can affect mitochondrial protein synthesis and lead to mitochondrial dysfunction.

Altogether, our findings, along with the evidence summarized above, strongly suggest that mtDNA plays a major role in the pathogenesis of atherosclerosis directly regulating the onset and progression of early vascular aging (16).

In patients with early-onset CAD, we hypothesize that the cumulative burden of traditional clinical risk factors may lead to more aggressive premature atherosclerosis through mechanisms such as oxidative stress and sustained inflammation, which are central to the progression of the mtDNA damage and dysfunction.

These observations raise significant clinical questions regarding broader preventive strategies to reduce the risk of premature coronary artery disease. First, our study confirms a high rate of concomitant modifiable cardiovascular risk factors in individuals with EOCAD (4–8), emphasizing the importance of increased efforts to reduce the burden of these risk factors and premature clinical events (11).

Second, ROC curve analysis suggested that both mitochondrial damage and dysfunction might be considered a biomarker reflecting the early diagnosis and prediction of EOCAD The evaluation of mtDNA could also serve as an easy and accessible biomarker that could be exploited for cardiovascular prognosis or for assessing responses to therapies (24). The incorporation of mitochondrial biomarkers into traditional risk assessments may have the potential to improve the accuracy of prediction. For example, it may help to reclassify intermediate risk patients into higher or lower risk categories. However, their integration into clinical practice will depend on standardization of measurement techniques and confirmation of their predictive value by robust evidence.

Third, targeting of mitochondrial dysfunction may be a feasible strategy to provide a more personalized approach to vascular risk managemen (38). Potential therapies include mitochondrial antioxidative therapies and lifestyle interventions (e.g., exercise, diet) aimed at mitigating mitochondrial dysfunction and ROS production. In addition, mitochondrial biogenesis enhancers may offer promising avenues for treating vascular damage caused by mitochondrial dysfunction by repairing mtDNA, such as increasing the activity of mitochondrial-specific enzymes (e.g., OGG1), or enhancing mitochondrial biogenesis. In addition, new gene-editing techniques such as CRISPR hold promise to correct mtDNA mutations (38).

Our study has some potential limitations. First, the sample size of patients is relatively small, which limits the precision of the associations. As result, the estimated ORs with the wide confidence intervals for risk factors should be interpreted cautiously. Second, the case-control study design comes with inherent sources of confounding and unmeasured biases. For instance, we did not measure family history or potential social and psychological biases. Third, the cohort comprised patients admitted to the hospital for coronary angiography, and some already had existing CAD or previous myocardial infarction and were receiving pharmacological treatment. Finally, mtDNA^4977^ deletion and mtDNA-CN were measured only at enrollment, without capturing potential variability over time. Indeed, there is potential uncertainty about the temporal variability of biomarkers when relying on measurements from a single point in time. Mitochondrial biomarkers may fluctuate with circadian rhythms, transient physiological states or in response to environmental exposures, which could affect their predictive utility. Future studies should, therefore, be focused on assessing mitochondrial biomarkers longitudinally in order to evaluate their temporal dynamics and improve the accuracy of risk prediction.

## 5 Conclusions

In conclusion, this study revealed that premature EOCAD is characterized by a high prevalence of modifiable cardiovascular risk factors and elevated mitochondrial damage and dysfunction. This underscores the need for more comprehensive efforts to reduce the burden of traditional risk factors and highlights the potential for innovative mitochondrial-targeted therapies to address vascular damage resulting from mitochondrial dysfunction.

## Data Availability

The raw data supporting the conclusions of this article will be made available by the authors, without undue reservation.
